# Structural basis of substrate diversity and functional evolution of archaeal RNA-splicing endonucleases

**DOI:** 10.1093/nar/gkaf845

**Published:** 2025-08-30

**Authors:** Yuna Miyata, Ryota Yamagami, Takuya Kawamura, Hiroyuki Hori, Akira Hirata

**Affiliations:** Department of Natural Science, Division of Science and Technology, Graduate School of Sciences and Technology for Innovation, Tokushima University, 2-1 Minamijosanjima-cho, Tokushima, Tokushima 770-8506, Japan; Department of Applied Chemistry, Graduate School of Science and Engineering, Ehime University, 3 Bunkyo-cho, Matsuyama, Ehime 790-8577, Japan; Department of Applied Chemistry, Graduate School of Science and Engineering, Ehime University, 3 Bunkyo-cho, Matsuyama, Ehime 790-8577, Japan; Department of Applied Chemistry, Graduate School of Science and Engineering, Ehime University, 3 Bunkyo-cho, Matsuyama, Ehime 790-8577, Japan; Department of Natural Science, Division of Science and Technology, Graduate School of Sciences and Technology for Innovation, Tokushima University, 2-1 Minamijosanjima-cho, Tokushima, Tokushima 770-8506, Japan

## Abstract

Maturation of transfer RNA molecules often requires removal of intronic sequences by endonucleases that recognize diverse RNA secondary structures. Archaeal splicing endonucleases [versatile RNA-splicing endonucleases (VSENs)] exhibit remarkable substrate versatility, yet the structural basis for this broad specificity has remained unclear. Here, we report the 1.8-Å crystal structure of ARMAN-2, an ϵ_2_-type VSEN from *Candidatus Micrarchaeum acidiphilum*, in complex with a synthetic bulge–helix–bulge RNA. The structure reveals that a lineage-specific insertion, the ARMAN-specific loop (ASL), interacts with the bulged region of the RNA and helps to orient the scissile phosphate for catalysis via conserved tyrosine and lysine residues. Functional assays confirmed the essential role of the ASL in substrate binding and cleavage. Structural comparisons with (αβ)_2_-type Crenarchaeal VSENs, which contain a distinct Crenarchaea-specific loop (CSL), and with a eukaryotic equivalent, the TSEN complex, which harbors a previously uncharacterized eukaryotic-specific loop (ESL), uncovered mechanistic convergence across domains of life. We show that the ESL occupies a position analogous to the ASL and CSL, and likely supports bulge stabilization in long introns. These findings establish a mechanistic model for broad substrate recognition by VSENs and suggest that loop-mediated RNA positioning co-evolved with intron complexity in archaeal and eukaryotic lineages.

## Graphical Abstract

**Graphical Abstract:**
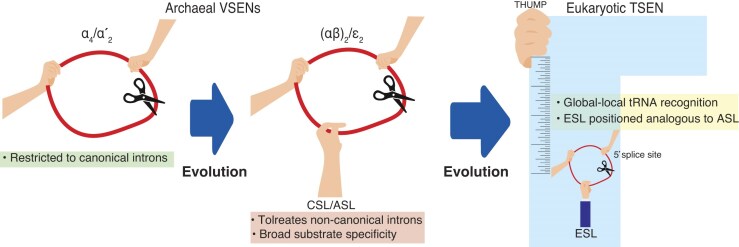

## Introduction

RNA splicing is a universally conserved mechanism that plays a central role in the maturation of precursor RNAs across all domains of life. In archaea and eukaryotes, the removal of introns from precursor transfer RNAs (tRNAs) is an essential step to generate functional tRNAs for translation. This reaction is carried out by a specialized ribonuclease, the RNA-splicing endonuclease [versatile RNA-splicing endonuclease (VSEN) in archaea, TSEN in eukaryotes), which cleaves the RNA backbone at precise exon–intron junctions [1–3].

Instead of the commonly used “EndA” to refer to the archaeal splicing endonuclease, we propose the term “VSEN” to reflect the ability of this enzyme to remove introns not only from pre-tRNAs, but also from pre-messenger RNAs [4–6] and ribosomal RNAs [7–9]. This is because, as a name, ‘EndA’ has been inconsistently applied across organisms, including unrelated nucleases, leading to confusion in the field [10]. The term “VSEN” emphasizes both the enzyme’s functional versatility and its evolutionary link with the eukaryotic TSEN [11].

Unlike eukaryotic TSENs that recognize the L-shaped tertiary structure of pre-tRNAs [12, 13], archaeal VSENs recognize and cleave introns based on specific RNA secondary structures [14]. The archaeal VSENs are classified into four types according to subunit composition, namely, homotetramer (α_4_), homodimer (α′_2_), heterotetramer (αβ)_2_, and homodimer [ϵ_2_: where ϵ represents the union of three units (α^N^–α–β^C^)] [15, 16]. The α and β subunits have been shown to be a catalytic and structural unit, respectively. Archaeal tRNA genes often contain introns at diverse and noncanonical positions—including in the D-arm, T-arm, and acceptor stem regions—rather than exclusively between positions 37 and 38 as in eukaryotes [14]. Examples include split tRNAs, permuted tRNAs, and multiple intron-containing tRNAs [17–20]. This diversity suggests that intron architecture in archaea has undergone significant evolutionary change [21, 22] and likely co-evolved with the substrate flexibility of VSENs [21, 23–25], which enables the accomodation of a wide range of RNA secondary structures and cleavage positions. The most well-characterized of these is the bulge–helix–bulge (BHB) motif, which forms a symmetrical structure that guides VSEN to its cleavage sites [14]. However, various studies have revealed that BHB-like structures—such as bulge–helix–loop (BHL) and other relaxed conformations—are also competent substrates for some VSENs.

Recent cryo-electron microscopy (cryo-EM) structures of the human TSEN complex bound to intron-containing pre-tRNAs have provided valuable insights into how this heterotetrameric endonuclease recognizes and processes eukaryotic intron substrates [26–29]. TSEN is composed of four subunits—TSEN2, TSEN15, TSEN34, and TSEN54—including two catalytic components (TSEN2 and TSEN34), which share structural and functional similarity with the α subunit of archaeal VSENs. Both VSEN and TSEN enable a catalytic mechanism reminiscent of RNase A, involving a triad of residues essential for phosphodiester bond cleavage [3, 30]. The human TSEN structures have also provided a framework for understanding the molecular basis of TSEN-related neurodevelopmental disorders, such as pontocerebellar hypoplasia [31–33]. These structures reveal how pathogenic mutations affect RNA binding and cleavage activities, thereby linking structural disruption to disease phenotypes. Furthermore, TSEN can process introns located within full-length pre-tRNAs that contain BHB or BHL motifs, as indicated by biochemical and structural studies. This supports the long-standing ‘molecular ruler’ model in which the geometry of the RNA substrate guides precise intron cleavage [12]. The shared functional and mechanistic features reinforce the evolutionary connection between archaeal and eukaryotic splicing endonucleases, suggesting that TSEN may have inherited its core catalytic framework and substrate recognition strategy from an ancestral VSEN-like enzyme [10, 24, 25].

Over 20 years ago, archaeal VSENs from Crenarchaea and Nanoarchaea were shown to cleave a variety of intron structures, revealing a broad substrate specificity [23, 34]. These studies led to the proposal that lineage-specific loops, such as the Crenarchaea-specific loop (CSL) [35–37] and a similar loop later identified in the ϵ_2_-type VSEN of the ultrasmall archaeon *Candidatus Micrarchaeum acidiphilum* (commonly referred to as ARMAN-2 [15, 38])—now termed the ARMAN-specific loop (ASL) [39]—play critical roles in relaxed substrate recognition. Both loops contain conserved lysine residues positioned near the active site, suggesting a shared mechanistic role not only in substrate anchoring but also—potentially—as catalytic residues directly contributing to RNA cleavage. Notably, archaeal species that encode VSENs with CSL or ASL motifs tend to harbor tRNA genes with highly diversified intron positions and structures, indicating a potential co-evolution between loop acquisition and intron variability [24]. Despite biochemical support for their involvement, the structural basis for how these loops contribute to broad substrate specificity—and whether this reflects evolutionary conservation or convergent adaptation—has remained unresolved.

We herein present the 1.8-Å high-resolution crystal structure of the ϵ_2_-type VSEN of ARMAN-2 in complex with an intron-containing RNA substrate. The structure reveals that the ASL forms direct hydrogen bonds and electrostatic interactions with the bulged region of the intron, stabilizing its conformation at the cleavage site. These findings provide the first structural evidence for loop-mediated substrate recognition in archaeal VSENs and uncover a conserved functional strategy shared with the eukaryotic TSEN.

## Materials and methods

### Protein expression and purification

Recombinant ϵ_2_-type VSEN of ARMAN-2 was expressed in *Escherichia coli* Rosetta 2 (DE3) cells (Novagen) using a pET-23b vector harboring the ARMAN-2 VSEN gene fused with a C-terminal hexahistidine tag [15]. The expression and purification procedure were based on a previously reported protocol [39], with slight modifications. Transformed cells were cultured in Luria-Bartani (LB) medium supplemented with 100-μg/ml ampicillin at 37°C for 4 h, until the cell density reached an OD_600_ of ∼0.8, then induced with 0.5-mM isopropyl β-D-1-thiogalactopyranoside (IPTG) and incubated for an additional 4 h at 37°C before harvesting by centrifugation. The supernatant was subjected to heat treatment at 50°C for 20 min to denature host proteins, followed by centrifugation. The clarified lysate was loaded onto a Ni–NTA Superflow column (Qiagen) pre-equilibrated with lysis buffer, and bound proteins were eluted with 500-mM imidazole. Eluted fractions were further purified on a HiTrap Heparin–Sepharose column (Cytiva) using a linear KCl gradient (50 mM–1 M) in buffer (20-mM Tris–HCl, pH 7.6, 10-mM 2-mercaptoethanol, 5% glycerol). Peak fractions were concentrated and subjected to gel filtration using a HiLoad 16/60 Superdex 75 column (Cytiva) in buffer (20-mM Tris–HCl, pH 7.6, 700-mM NaCl, 10-mM 2-mercaptoethanol, 5% glycerol). Protein purity was checked by sodium dodecyl sulphate–polyacrylamide gel electrophoresis (Supplementary Fig. S1). Mutant variants, including Y160A, Y160F, K161A, K161R, Y160A/K161A, and ΔASL, were constructed to investigate the functional contributions of ASL residues. Point mutants were generated using the QuickChange site-directed mutagenesis kit (Stratagene), while the ΔASL deletion mutant was created using reverse polymerase chain reaction (PCR). Primer sequences for each mutant are listed in Supplementary Table S1. All constructs were verified by DNA sequencing and were used for protein expression and purification following the same protocols as for wild-type VSEN.

### Crystallization

Purified ARMAN-2 VSEN was concentrated to 10–15 mg/ml in buffer containing 20-mM Tris–HCl, pH 7.6, and 200-mM NaCl. A synthetic 21-mer BHB RNA duplex was used for complex formation. The RNA sequence was based on that employed in the crystallographic study of the *Archaeoglobus fulgidus* (AFU) α′_2_-type VSEN–RNA complex [3]. It consists of the following strands: 5′-AAUGCAGCGGUCAAAGGUCG-3′ and 5′-AAUCGACCGACCAdUAGCUGCA-3′, where dU represents a 2′-deoxyuridine introduced at position 14 to prevent cleavage. The two strands were annealed at equimolar concentrations in annealing buffer (10-mM Tris–HCl, pH 7.6, 100-mM NaCl, 10-mM MgCl_2_) by heating at 60°C and slowly cooling to room temperature. The protein–RNA complex was prepared at a 1:1.2 molar ratio and incubated at 20°C for 30 min. Crystallization conditions were determined by the hanging-drop vapor diffusion method at 20°C using Crystal Screen reagent kits (Hampton Research). Each drop consisted of 1 μl of complex solution mixed with 1 μl of reservoir solution and equilibrated against 200 μl of reservoir solution. Initial crystals appeared within 3–5 days and were optimized using Grid Screens (Hampton Research). Crystals suitable for X-ray diffraction appeared in a condition containing 2.0-M ammonium sulfate, 0.1-M sodium acetate trihydrate pH 4.5. The obtained crystals were cryoprotected by briefly soaking in crystallization reagent supplemented with 25% (v/v) glycerol before flash-cooling in liquid nitrogen.

### Data collection and structure refinement

X-ray diffraction data for ARMAN-2 VSEN–RNA crystals were collected at 100 K on beamline BL38B1 at the SPring-8 synchrotron radiation facility (Hyogo, Japan). Diffraction images were processed and scaled using the HKL2000 package [40]. The structure was solved by molecular replacement with Phaser [41] using the coordinates (PDB ID: 4FZ2) and reduced data of the previously reported apo-form of ARMAN-2 VSEN [39], assuming space group *P*1. Two VSEN molecules and one RNA duplex were found in the asymmetric unit. Model building was carried out manually using Coot [42] based on the initial electron density map. The RNA duplex used exhibited pseudo-symmetry in its sequence and structure, with similar features at both the 5′ splice site (5′SS) and 3′ splice site (3′SS). To distinguish the two orientations, the structure was refined in space group P1, which allowed explicit modeling of the RNA strands without imposing crystallographic symmetry constraints. This approach enabled unambiguous modeling of each RNA strand, and all nucleotides were refined with full (100%) occupancy. Refinement was then performed with Refmac5 in the CCP4 suite [43]. The final model was refined to a resolution of 1.80 Å, with *R*-work and *R*-free values of 18.8% and 23.1%, respectively (Table 1). The refined model includes residues 2–387 of chain A and residues 2–385 of chain B of VSEN, one 13-base pair RNA duplex (composed of two 13-nt strands spanning residues 6–18), 349 water molecules, and 4 acetic acid ions. The geometry of the final structure was validated using PROCHECK [44]. Ramachandran plot statistics showed that 92.1% of residues were in the most favored regions, 7.6% in additionally allowed, 0.3% in generously allowed regions (Table 1). To visualize unbiased electron density for the RNA substrate, we generated omit maps using the phenix.polder tool [45] implemented in the Phenix software suite [46]. In each case, the coordinates of the RNA molecule were removed from the model, and bulk solvent masking was locally excluded in the omitted region to reduce density flattening. Polder maps were calculated both for the entire RNA substrate and separately for the bulged nucleotides at the 5′SS and the 3′SS. The resulting maps were contoured at 2.0σ or 2.5σ for preparation of the omit map panels shown in Supplementary Fig. S2. The refined coordinates and structure factors have been deposited in the Protein Data Bank under accession code 9W2K. All structural figures were generated by CCP4mg [47].

**Table 1.** Data collection and refinement statistics

	ϵ2 VSEN–RNA
*Data collection*	
Space group	*P*1
Cell dimensions	
a,b,c (Å)	53.514, 65.648, 71.802
α,β,γ (°)	114.46, 111.89, 90.01
Resolution (Å)	50–1.80 (1.86–1.80)
Rmerge^a^	5.5 (26.1)
*I/σI*	26.3 (3.3)
Completeness (%)	97.1 (95.9)
Redundancy	2.3 (2.3)
	
*Refinement*	
Resolution	33.53–1.80
No. reflections	69 302
*R* work^b^*/R* free^c^	18.76/23.10
No. atom	7209
Protein	6288
Nucleic acid	556
Acetic acid ion	16 (acetate × 4)
Water	349
Avg. B-factors (Å2)	24.68
RMSD^d^	
Bond lengths (Å)	0.008
Bond angles (°)	1.57
Ramachandran plot (%)	
Most favored	92.1
Additional allowed	7.6
Generously allowed	0.3
Disallowed	0.0

The value in the parentheses is for the highest resolution shell.

^a^Rmerge = ΣΣj|<I(h)> – I(h)j|/ΣΣj|<I(h)>|, where < I(h)> is the mean intensity of symmetry-equivalent reflections.

^b^Rwork = Σ (IIFp(obs) – Fp(calc)II)/ΣIFp(obs)I.

^c^Rfree = R factor for a selected subset (5%) of reflections that was not included in earlier refinement calculations.

^d^RMSD, root-mean-square deviation.

### Intron-cleavage activity

Intron-cleavage assays were carried out as previously described [39], with modifications in transcription product purification, reaction volume, and time-course sampling. Precursor RNAs corresponding to ARMAN-2 pre-tRNA^Ile^ (UAU) and pre-tRNA^Cys^ (GCA) were synthesized by *in vitro* transcription using T7 RNA polymerase. Transcripts were purified by phenol–chloroform extraction followed by ethanol precipitation. Cleavage reactions were performed in a total volume of 24 μl in reaction buffer containing 50-mM Tris–HCl, pH 7.6, 200-mM KCl, 5-mM MgCl_2_, and 6-mM 2-mercaptoethanol. Each reaction contained 0.3-nmol RNA substrate and 3 μl of VSEN (0.01 nmol wild type or mutant). Incubation was performed at 50°C, and 9-μl aliquots were removed at designated time points. For the ARMAN-2 pre-tRNA^Ile^ (UAU) substrate, samples were collected at 0 and 30 min; for pre-tRNA^Cys^ (GCA), at 0 and 5 min. In the case of the ΔASL mutant, the total reaction volume was doubled (48 μl), and 9-μl aliquots were taken at 0, 30, 60, and 120 min. Reaction products were separated by 15% denaturing polyacrylamide gel electrophoresis containing 7-M urea. Gels were stained with 0.05% toluidine blue and analyzed to evaluate cleavage activity based on the appearance of product bands. All assays were performed in at least three independent experiments to ensure reproducibility.

## Results and discussion

### Overall structure of the ARMAN-2 VSEN–RNA complex

**Figure 1.**
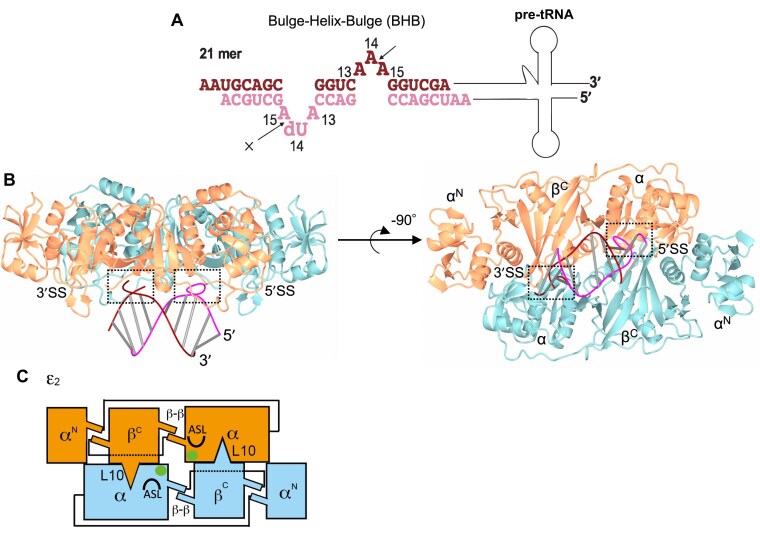
Crystal structure of the ARMAN-2 VSEN–RNA complex containing a BHB motif. (**A**) Primary sequence and predicted secondary structure of the synthetic pre-tRNA used for co-crystallization. The RNA contains a canonical BHB motif, with the 5′SS and 3′SS indicated by arrows. The 5′SS contains a 2′-deoxyuridine at position 14 (dU14) which prevents cleavage at this site (as marked by a cross). In the sequence representation, nucleotides belonging to the RNA strand containing the 5′SS are shown in pink, and those belonging to the strand containing the 3′SS are shown in dark red. (**B**) Overall structure of the ARMAN-2 VSEN–RNA complex. The ϵ_2_-type VSEN forms a homodimer, with the two monomers colored cyan and orange, respectively. The RNA duplex is rendered as a line diagram, with the strand containing dU14 highlighted in pink and the complementary strand in dark red. Dashed boxes indicate the locations of the 5′SS and 3′SS. The three structural elements comprising the ϵ subunit—α^N^, α, and β^C^—are labeled. (**C**) Schematic representation of the ϵ_2_-type VSEN homodimer. The two subunits are shown in cyan and orange. Key dimerization features, including the L10 loop and β–β interactions, are indicated. Green spheres mark the locations of the catalytic centers, with the ASL also depicted.

To elucidate the structural basis for the broad substrate specificity of ARMAN-2 VSEN, we sought to determine its high-resolution crystal structure in complex with RNA. Despite extensive crystallization trials using a range of synthetic RNA oligonucleotides—including canonical BHB motifs as well as relaxed BHB-like conformations with disrupted bulge regions at the 5′SS or 3′SS—no co-crystals could be obtained. We therefore employed the same synthetic BHB RNA duplex previously used for the crystallographic analysis of the AFU α′_2_-type VSEN–RNA complex [3] (Fig. 1A). This RNA forms a symmetric BHB motif and includes a 2′-deoxyuridine at position 14 (dU14) to prevent cleavage at the 5′SS. Using this RNA, we successfully obtained crystals of ARMAN-2 VSEN in complex with RNA and collected high-resolution X-ray diffraction data. The structure was solved by molecular replacement using the apo form of ARMAN-2 VSEN as the search model and refined to 1.8-Å resolution. The asymmetric unit contained two ϵ protomers of ARMAN-2 VSEN and a 13-nucleotide RNA duplex. The electron density map for the RNA was exceptionally well resolved, allowing unambiguous model building of the substrate, as further confirmed by polder omit maps (Supplementary Fig. S2). Similar to the RNA in the AFU VSEN complex [3], the duplex in our structure exhibits pseudo-symmetry at the sequence and structural levels. However, by reprocessing the diffraction data in space group *P*1, we were able to explicitly model each RNA strand without imposing crystallographic symmetry constraints, and all nucleotides were refined with full (100%) occupancy. Superposition of chain A and chain B in the *P*1 structure yielded a root-mean-square deviation (RMSD) of 0.06 Å over 384 aligned Cα atoms, indicating that the two protomers are virtually identical in conformation. Figure 1B shows the overall structure of the ARMAN-2 VSEN–RNA complex. Within the crystal lattice, two ϵ protomers and a 13-nt RNA duplex are present in the asymmetric unit. A functionally relevant ϵ_2_ homodimer is generated through crystallographic symmetry, involving conserved β–β interactions and L10 loop contacts (Fig. 1C). The two active sites and ASLs are symmetrically positioned at the dimer interface, with the RNA duplex bound centrally across the homodimer surface. As will be discussed in detail, a characteristic bulged conformation containing the noncleavable dU14 is observed at the 5′SS, representing the pre-cleavage state. In contrast, the 3′SS features a 2′,3′-cyclic phosphate group, indicating that cleavage has occurred at this site.

Supplementary Figure S3A shows the structural superposition of ARMAN-2 VSEN in its apo form and in complex with RNA, revealing a high degree of similarity (RMSD = 0.93 Å over 387 Cα atoms). These findings suggest that the enzyme adopts a pre-formed conformation for RNA recognition, with only localized conformational changes occurring upon substrate binding. Notably, a minor conformational change occurs in the catalytic center: in the apo structure, the loop containing H251 adopts a 3_10_-helix conformation, whereas in the complex, this helix is lost and the H251 side chain stacks against the third bulged nucleotide (A15) at the 5′SS (Supplementary Fig. S3B). Furthermore, the first bulged nucleotide (A13) is stabilized through a π–cation interaction involving R275 and W384 from the adjacent protomer with both residues being slightly shifted upon association with the RNA (Supplementary Fig. S3C).

### RNA recognition and cleavage by ARMAN-2 VSEN

**Figure 2.**
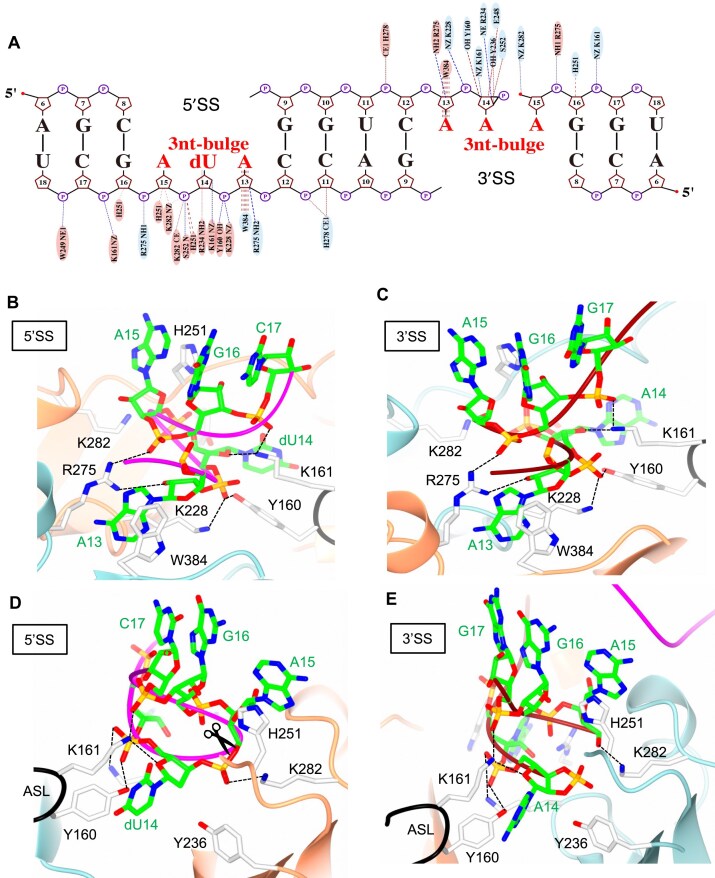
Recognition and cleavage of BHB RNA by ARMAN-2 VSEN. (**A**) Residues contacting the BHB RNA were diagrammed using Nucplot [49] and are color-coded as in Fig. 1. (**B, C**) Recognition of the bulged region at the 5′SS (**B**) and 3′SS (**C**), highlighting π–cation interactions between nucleotide A13 and residues R275 and W384. (**D, E**) Detailed views of the active site poised for cleavage at the 5′SS (**D**) and after cleavage at the 3′SS (**E**). In panels (B)–(E), RNA is shown as sticks with colors indicative for constituting atoms: carbon (green), nitrogen (blue), oxygen (red), and phospor (yellow); protein residues involved in RNA recognition or catalysis are shown as light gray sticks with nitrogen and oxygen atoms in blue and red, respectively. Residues located on the ASL (black), including Y160 and K161, are also shown in light gray. Hydrogen bonds are represented by dotted lines, and the intron cleavage site is marked by a scissor in panel (D). Other structural elements are colored as in Fig. 1B.

To gain insight into how ARMAN-2 VSEN recognizes its RNA substrate and catalyzes intron cleavage, we analyzed the interactions between the protein and the bound BHB RNA duplex (Fig. 2A). Residues involved in RNA binding are predominantly localized around the central bulge region of the BHB motif, underscoring their critical role in substrate recognition and catalysis. Outside of this region, RNA density was largely disordered and not modeled. A detailed list of hydrogen bonds and van der Waals contacts between RNA and the protein is provided in Supplementary Table S2. Figure 2B captures the pre-cleavage state at the 5′SS, where the first bulged nucleotide, A13, is sandwiched between R275 and W384 from the adjacent protomer via π–cation interactions. Similar interactions involving Arg and Trp residues have been proposed previously for ϵ_2_- and (αβ)_2_-type VSENs [35, 36, 39] but our structure is the first to visualize them directly. In α_4_- and α′_2_-type VSENs, however, two arginine residues are involved in base recognition at this position. In the ARMAN-2 VSEN–RNA complex, R275 (NH1) forms hydrogen bonds with the 2′-OH of A13 and the phosphate of G16, thus likely stabilizing the bent conformation of the RNA. Additional stabilization of the bulge is provided by K228, which forms a hydrogen bond with the phosphate of dU14. Comparable interactions of R275 and K228 occur at the 3′SS in the post-cleavage state (Fig. 2C). These findings mirror the interactions seen in the AFU α′_2_-VSEN–RNA complex in which R280, the residue corresponding to R275, has a similar role as R275, suggesting conservation of function. Figure 2D and E show alternative views of the catalytic centers at the 5′SS and 3′SS, respectively. The three conserved catalytic residues—Y236, H251, and K282—are positioned around the scissile phosphate. Y236 is oriented toward the ribose of dU14 and is ideally placed to abstract the proton from the 2′-OH group, facilitating the in-line nucleophilic attack. H251 undergoes a local conformational rearrangement upon RNA binding (Supplementary Fig. S3B) and stacks with A15, positioning its Nδ1 atom near the leaving 5′-oxygen. This conformational change likely activates H251 for catalysis. Despite its mechanistic relevance, the structural transition of H251 has received limited attention in prior studies, suggesting a novel role for the third bulged nucleotide in triggering the catalytic conformation. This observation may explain why AFU α′_2_-VSEN requires a strict 3-nt bulge, whereas broader specificity is associated with the (αβ)_2_-type VSEN from *Aeropyrum pernix* (APE) [36]. In the ARMAN-2 VSEN–RNA complex, the NZ atom of K282 and the amino group of S252 form a hydrogen bond with the nonbridging phosphate oxygen of A15, possibly stabilizing the transition state during cleavage. This interaction is comparable to that of K287 and S258 in AFU-VSEN and is consistent with its role in facilitating in-line attack, as previously described [3]. Importantly, residues Y160 and K161 in the ASL of ARMAN-2 VSEN may also contribute to catalysis and substrate stabilization. Y160 forms hydrogen bonds with the phosphate of dU14, while K161 makes contacts with the ribose O4′ of dU14 and the phosphate of C17. These interactions suggest dual roles for the ASL in both anchoring the RNA and fine-tuning the geometry of the scissile phosphate, enabling ARMAN-2 VSEN to process a broad range of substrates.

At the 3′SS, the phosphodiester bond between A14 and A15 is cleaved, and A14 harbors a 2′,3′-cyclic phosphate group, consistent with the cleavage mechanism [3, 30] (Fig. 2E). While the positioning of K282 remains consistent, other residues retain conformations similar to those observed at the 5′SS, reinforcing the catalytic symmetry of the dimer. Together, these findings support a model in which the conserved catalytic residues and the ASL cooperatively ensure precise intron cleavage. K161 in particular plays a central role by interacting with both the ribose and phosphate backbone of the RNA, suggesting that the ASL contributes not only to RNA anchoring but also to catalysis. Additionally, the proximity of Y160 to the bulged region and its potential hydrogen bonding with the phosphate group of the central nucleotide of the bulge (A14, dU14) imply that Y160 may further stabilize the RNA conformation required for efficient cleavage.

### Intron-cleavage activity of ASL mutants

**Figure 3.**
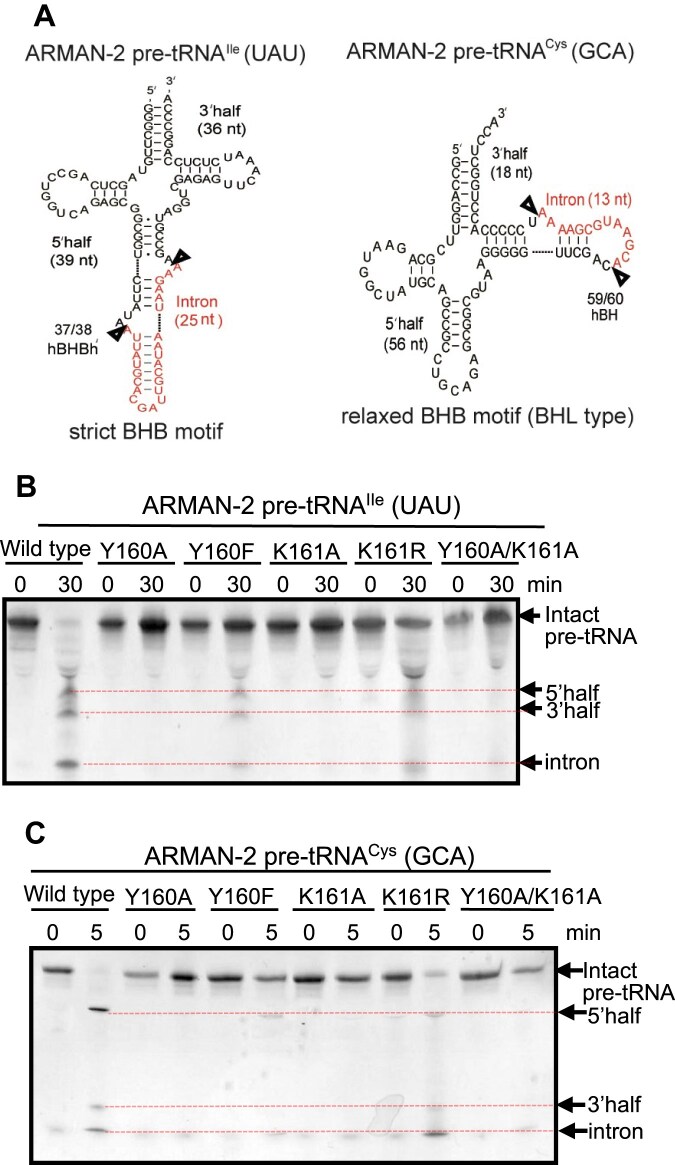
Cleavage activity of wild-type and ASL mutants of ARMAN-2 VSEN. (**A**) Predicted secondary structures of ARMAN-2 pre-tRNAs used as substrates. The left panel depicts pre-tRNA^Cys^ (GCA) and the right panel depicts pre-tRNA^Ile^ (UAU). Arrows indicate the expected splice sites at the exon–intron boundaries. (**B**) Cleavage activity of the wild type and five ASL mutants (Y160A, Y160F, K161A, K161R, and Y160A/K161A) using pre-tRNA^Ile^ (UAU) as a substrate. (**C**) Cleavage activity of the same enzyme variants using pre-tRNA^Cys^ (GCA). Reaction mixtures were separated by 15% polyacrylamide/7 M urea gel electrophoresis. Cleavage products (5′ exon, intron, and 3′ exon) are indicated by arrows on the right side of each gel image, with red dotted lines extending from the arrow tips to highlight the corresponding bands.

To investigate the functional importance of the ASL in catalysis and substrate recognition, we performed intron-cleavage assays using a series of ASL mutants and two RNA substrates under identical *in vitro* conditions. By testing a BHB-type (pre-tRNA^Ile^) and a BHL-type (pre-tRNA^Cys^) substrate we could examine whether the effects of mutations were consistent across structurally distinct introns (thereby addressing the ability of the recombinant proteins to efficiently cleave multiple classes of intron-containing RNAs) (Supplementary Table S1 and Fig. 3A). As previously shown [39], wild-type ARMAN-2 VSEN efficiently cleaves both substrates under standard assay conditions (Fig. 3B and C). In contrast, alanine substitutions at Y160, K161, or both (Y160A, K161A, Y160A/K161A) substantially impaired cleavage activity, with no detectable cleavage products observed under the assay conditions for either RNA substrate, while more conservative substitutions (Y160F and K161R) retained partial activity, indicating that both the aromatic and basic character of these residues are functionally relevant. These results extend those of our earlier study [39], in which alanine substitutions of the three conserved catalytic residues (Y236, H251, and K282) abolished cleavage activity. Consistent with our structural analyses, the findings indicate that catalysis depends only rely on direct participation of these three residues but also on ASL residues Y160 and K161, which play auxiliary yet essential roles in facilitating efficient catalysis.

To further clarify the contribution of the ASL, we tested the effect of complete removal of this loop (ΔASL). Cleavage activity of the ΔASL mutant was substantially reduced for both substrates. With the BHB-type pre-tRNA^Ile^ (Supplementary Fig. S4A), cleavage products gradually emerged after 30 min and were clearly detectable at 120 min, while for pre-tRNA^Cys^ (Supplementary Fig. S4B), only faint signals corresponding to 3′-half fragments were detectable, with no obvious accumulation over time of an intron fragment. This suggests that deletion of the ASL impairs the cleavage reaction, particularly at the 5′SS, and reduces the ability of the enzyme to process structurally diverse substrates. Notably, extended incubation led to nonspecific RNA degradation, making interpretation of the results more difficult; however, the near-complete loss of specific cleavage activity strongly supports a critical functional role for the ASL. Structural analysis provides a mechanistic rationale for these observations. At the 5′SS, K161 forms hydrogen bonds with the ribose O4′ of dU14 and the phosphate of C17, which likely stabilizes the scissile phosphate and fixes the RNA in a position required for cleavage. The K161R mutant retained partial activity, consistent with the requirement for a positively charged side chain. Tyr160, positioned adjacent to Lys161, also forms a hydrogen bond with dU14 OP1 and appears to restrict the orientation of the lysine side chain. This interaction may be important for aligning K161 in order to make productive contacts with the RNA backbone. Together, these residues may act in concert to secure the bulged region of the intron in place, allowing precise cleavage by the three conserved catalytic residues. These findings support the notion that the ASL not only contributes to RNA binding but also promotes catalytic efficiency and broad substrate specificity. In particular, the cooperative function of Y160 and K161 may represent a unique adaptation in ARMAN-2 VSEN, enabling it to recognize and process diverse intron structures with high specificity and fidelity.

### Structural comparison with the AFU α′_2_-type VSEN–RNA complex

**Figure 4.**
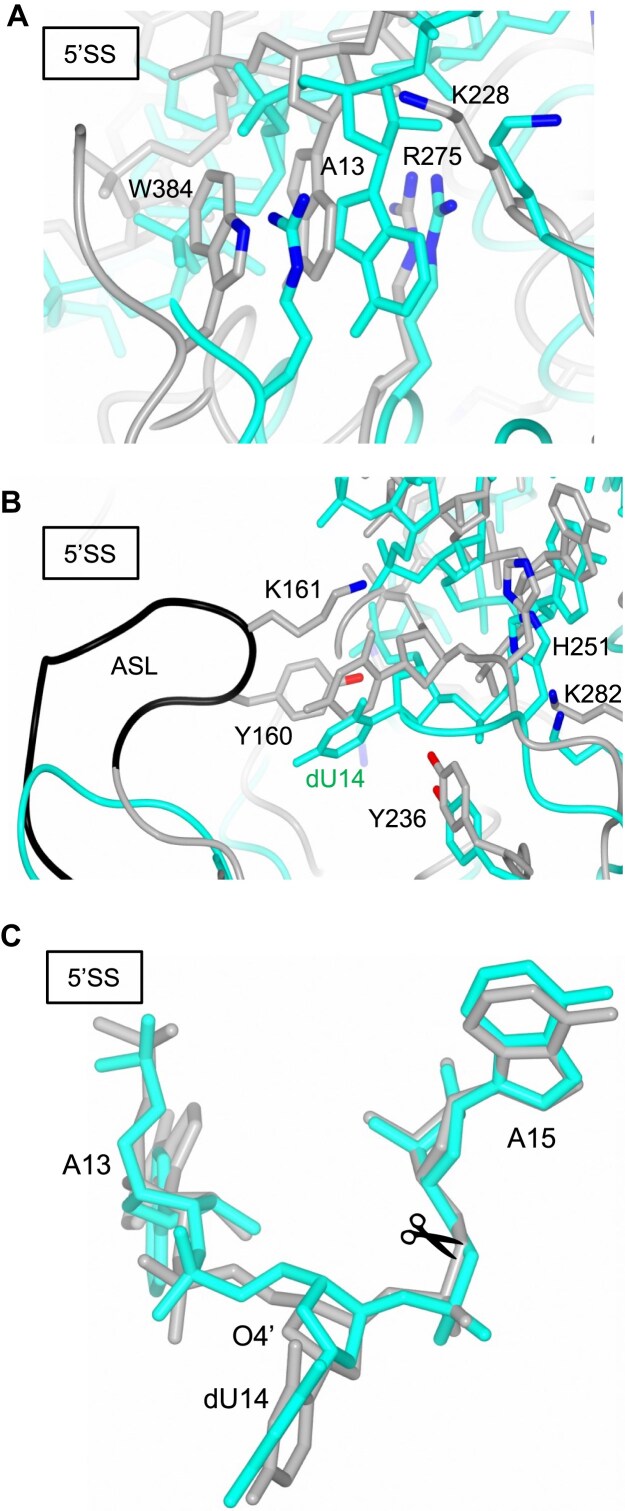
Structural comparison of ARMAN-2 ϵ_2_-type VSEN–RNA and AFU α′_2_-type VSEN–RNA complexes. (**A**) Close-up view of the 5′SS highlighting π–cation interactions with the bulged base A13. In the ARMAN-2 VSEN complex (cyan), R275 and W384 are depicted as stick models with nitrogen atoms in blue; in the AFU α′_2_-type VSEN complex (PDB ID: 2GJW, dark gray), the corresponding residues are likewise depicted. (**B**) Detailed comparison of the catalytic center at the 5′SS. Conserved catalytic residues in ARMAN-2 VSEN (Y236, H251, K282) and their counterparts in the α′_2_-type VSEN are depicted as in panel (A) with oxygen atoms in red. For Y160 and K161, located on the ASL of ARMAN-2 VSEN (black), the carbon atoms are depicted in dark gray. (**C**) Superposition of the bulged region containing dU14 at the 5′SS from both complexes. The RNA backbones are illustrated with the scissile phosphate groups of A15 at the intron cleavage sites marked by a scissor.

To elucidate structural features that may contribute to the broad substrate specificity of ARMAN-2 VSEN, we compared its RNA-bound structure with that of the α′_2_-type VSEN from AFU the specifity of which is limited to canonical BHB containing substrates [16, 23]. The overall fold of the two enzyme–RNA complexes is highly similar, as demonstrated by the structural alignment of 211 Cα atoms, which yielded an RMSD of 3.00 Å (Supplementary Fig. S5A). This overlap highlights the evolutionary conservation of the core architecture and dimerization mode between ϵ_2_-type and α′_2_-type VSENs, despite differences in substrate range. Detailed comparison at the 5′SS revealed that both enzymes stabilize the first bulged nucleotide of the intron via π–cation interactions. In the ARMAN-2 complex, this stabilization is mediated by R275 and W384 on the opposing protomer of the homodimer (Fig. 2A–C), forming a stacking interaction with the adenine base of A13 (Fig. 4A). In contrast, in the AFU α′_2_-type VSEN complex two arginine residues from within the same protomer convey a similar mode of bulge recognition. As discussed above, R275 (or its AFU equivalent R280) also interacts through a hydrogen bond with the RNA backbone (Fig. 2B and C), contributing to the stabilization of the bent conformation of the bulged region. Notably, in AFU VSEN, the R280A mutant abolishes intron-cleavage activity [3], underscoring the essential functional role of this arginine residue. In contrast, while W384 in ARMAN-2 VSEN participates in π–cation interactions with the bulged base, this position is more variable across species and can be functionally replaced by an arginine in other VSEN homologs [39]. This suggests that a positively charged residue is indispensable at the R275-equivalent position for bulge stabilization, whereas the corresponding Trp/Arg site may have evolved with greater flexibility, likely reflecting functional redundancy in π-stacking contributions. Figure 4B provides a detailed comparison of the catalytic centers at the 5′SSs of the ARMAN-2 and AFU VSEN–RNA complexes. The three essential catalytic residues in ARMAN-2 VSEN—Tyr236, His251, and Lys282—are spatially conserved and adopt similar conformations to their counterparts in AFU VSEN. Previous studies have shown that alanine substitutions of these residues abolish intron-cleavage activity in both enzymes [3, 39], underscoring their indispensable catalytic roles. The major structural distinction between the two enzymes lies in the presence of the ASL, which is absent in AFU VSEN. The introduction of the ASL into AFU VSEN conferred broad substrate specificity to the mutant enzyme [39], demonstrating that the ASL plays a functional role in substrate accommodation. However, deletion of the ASL from ARMAN-2 VSEN (ΔASL) not only severely compromised its ability to process two different RNA substrates but also dramatically impaired its general intron-cleavage activity—even against canonical BHB motifs (Supplementary Fig. S4). These observations suggest that ASL is not merely an auxiliary recognition element but is also essential for catalytic function in ARMAN-2 VSEN. To explore the structural basis for how ASL deletion results in loss of activity despite the preservation of catalytic residues, we compared the 5′SS regions of the ARMAN-2 and AFU complexes by superimposing only the bound RNA (Supplementary Fig. S5B). The RMSD of 0.86 Å across 80 phosphate backbone atoms indicates that the overall architecture of the BHB motif is largely preserved. Nevertheless, Fig. 4C highlights a critical local divergence: the phosphate group of A13 and the ribose moiety of dU14 are significantly displaced in ARMAN-2 VSEN relative to the AFU structure. Specifically, the distance between the O4′ atoms of dU14 in the two complexes is 1.4 Å, suggesting that ASL—through hydrogen bonding between K161 and dU14 O4′—stabilizes the ribose conformation at the 5′SS. This observation supports the idea that K161, assisted by Y160, precisely positions the scissile phosphate for in-line nucleophilic attack, thereby facilitating catalysis. The absence of ASL likely prevents proper anchoring of the intron bulge, disrupting substrate alignment with the catalytic residues, which results in catalytic incompetence. In contrast, AFU VSEN achieves productive positioning of the RNA substrate using conserved residues, an arginine 280 for bulge recognition and a lysine 287 for in-line attack stabilization, albeit with more limited substrate flexibility [16, 23]. Taken together, these structural insights suggest that the ASL in ARMAN-2 VSEN acts as a molecular guide that tunes the orientation of the substrate’s scissile phosphate, enabling efficient and versatile intron cleavage. This loop, therefore, is not merely an accessory feature but a critical determinant of both catalytic activation and broad substrate specificity.

### Structural and evolutionary parallels between ASL and CSL

To explore the possibility that the ASL-mediated recognition mechanism observed for ARMAN-2 VSEN has been evolutionarily conserved, we compared the structure of ARMAN-2 VSEN with that of other archaeal endonucleases thereby focusing on Crenarchaeal VSENs that possess a CSL. Structural superposition revealed that the ASL and CSL occupy analogous positions near the catalytic center, and in both cases, a conserved lysine residue is positioned so that this residue interacts with the RNA substrate (Supplementary Fig. S6A).

Building on the structural resemblance observed between the ASL and CSL, we further examined their sequence conservation and the potential functional convergence across archaeal species. The ASL in ARMAN-2 VSEN consists of 11 amino acids, and sequence alignment of this loop to the homologous regions in VSENs of four other *Candidatus* species revealed that not only K161 but also Y160 is strictly conserved (Supplementary Fig. S6B). This conservation strongly suggests that both residues are essential for the function of the ASL. In contrast, the CSL found in Crenarchaeal VSENs is more diverse with respect to length, ranging from 15 to 18 residues—4–7 residues longer than the ASL. As illustrated in Supplementary Fig. S6A, the CSL appears structurally larger than the ASL, possibly due to the absence of steric hindrance that may be introduced by a loop formed by I243–E248 in ARMAN-2 VSEN. Despite these differences, both CSL and ASL loops possess a lysine residue positioned near the catalytic center. In CSL, this lysine is typically followed by a proline or valine, and then by another conserved positively charged residue such as lysine or arginine (Supplementary Fig. S6C). In the (αβ)_2_-type VSEN from APE, substitution of these residues (P45A and R46A) drastically reduced intron-cleavage activity [36]. P45 likely contributes to loop stability, while R46 may interact with the RNA phosphate backbone downstream of the bulge to maintain CSL structure. This arrangement would parallel the ASL conformation in ARMAN-2 VSEN, where Y160 is thought to stabilize the side chain of K161. This stabilization may facilitate the optimal positioning of K161 for hydrogen bonding with the ribose O4′ and C17 phosphate, thereby promoting precise scissile phosphate alignment. Similarly in APE CSL, R46 might orient K44 to interact with the RNA, mirroring the functional coupling of Y160 and K161 in ASL. Because K44 and a conserved positively charged residue (either Arg or Lys) at position 46 are conserved across other Crenarchaeal VSENs, it is plausible that CSLs universally contribute to accurate intron cleavage and possibly broader substrate recognition. Despite low sequence identity, the conserved lysine residue and the similarity of the overall spatial configurations support the idea that ASL and CSL motifs have undergone convergent evolution with the outcome that these loops fulfill similar roles in catalysis and RNA substrate positioning.

### Evolutionary insights from structural comparison of archaeal and eukaryotic splicing endonucleases

**Figure 5.**
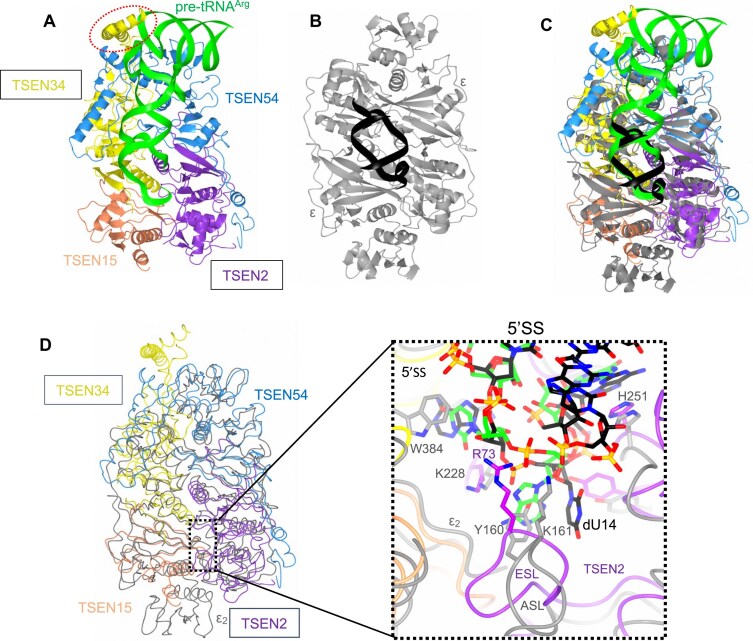
Structural comparison of the ARMAN-2 ϵ_2_-type VSEN–RNA and human TSEN–pre-tRNA^Arg^ (UCU) complexes. (**A**) Ribbon diagram of the human TSEN–pre-tRNA^Arg^ (UCU) complex (PDB ID: 8ISS). The heterotetrameric human TSEN is composed of TSEN2 (purple), TSEN15 (orange), TSEN34 (yellow), and TSEN54 (cyan). Catalytic subunits TSEN2 and TSEN34 are boxed. The THUMP domain of TSEN34 is highlighted with a red dashed outline. The tRNA**^Arg^** molecule is shown in green. (**B**) Ribbon diagram of the ARMAN-2 ϵ_2_-type VSEN–RNA complex. The ϵ protomer is shown in gray and the RNA duplex in black. (**C**) Superimposition of the two complexes based on 159 equivalent Cα atoms (RMSD = 2.28 Å). (**D**) Left panel: Line diagram of the overlay shown in panel (C), highlighting the 5′SS (dashed box). Right panel: Close-up view of the 5′SS. Key residues from each subunit are depicted as stick models and colored as in panels (A) and (B); RNA nucleotides are shown as stick models colored as in Fig. 2 with carbon in green (for tRNA^Arg^) or in black [in the case of the RNA duplex of panel (B)]. The ESL occupies a position similar to that of the ASL in the ARMAN-2 VSEN.

**Figure 6.**
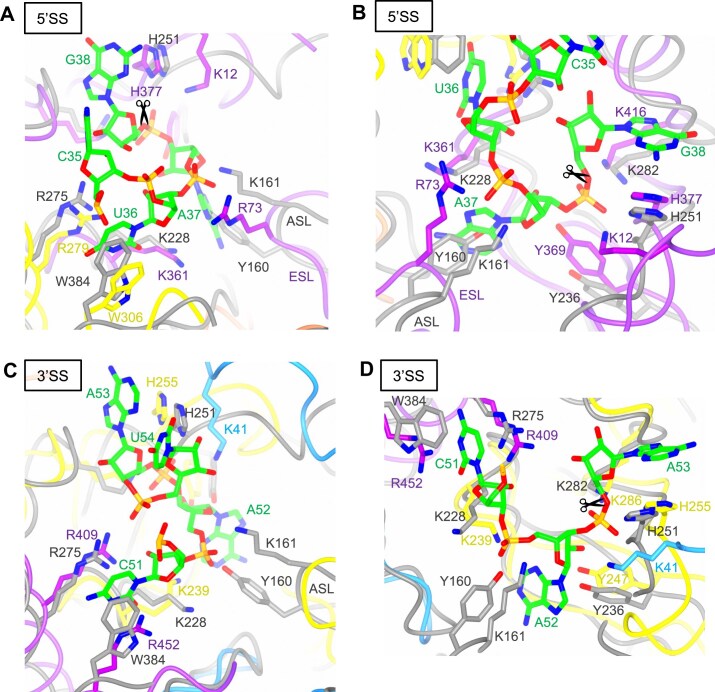
Structural comparison of the 5′SS and 3′SS of the ARMAN-2 VSEN–BHB RNA and human TSEN–pre-tRNA^Arg^ (UCU) complexes. (**A**-**D**) Close-up views from different angles of the catalytic center at the 5′SS (**A**, **B**) and 3′SS (**C**, **D**) showing the first nucleotide of the bulge [(U36 in panels (A) and (B); C51 in panels (C) and (D)] and its π–cation interaction with surrounding residues. Key residues from each subunit are depicted as stick models colored as in Fig. 5 with the carbon in yellow (TSEN34), purple (TSEN2), cyan (TSEN54), orange (TSEN15), or gray (ARMAN-2 VSEN); the complexed tRNA^Arg^ is colored as in Fig. 2 with the carbon in green, while the BHB RNA duplex bound to ARMAN-2 VSEN is not shown. The intron cleavage site is indicated in panels (A), (B), and (D) by a scissor. (PDB ID: 8ISS for human TSEN-–pre-tRNA^Arg^ (UCU) complex).

Four independent research groups have recently resolved the cryo-EM structures of human TSEN complexes bound to pre-tRNA substrates [26–29]. These landmark studies have revealed the previously elusive mechanism by which TSEN recognizes intron-containing tRNAs and identified residues critical for catalysis that were altered by disease-associated mutations [31–33, 48]. To investigate the mechanistic similarities and differences in RNA recognition and intron cleavage between archaeal and eukaryotic splicing endonucleases, we compared the structure of the ARMAN-2 VSEN–RNA complex with that of the human TSEN complex bound to pre-tRNA^Arg^ (UCU) (Fig. 5A). The human TSEN forms a heterotetramer comprising two catalytic subunits (TSEN2 and TSEN34) and two noncatalytic subunits (TSEN15 and TSEN54), while ARMAN-2 VSEN functions as a homodimer composed of two ϵ protomers (Fig. 5B). Despite this difference in overall subunit composition, structural superposition revealed a well-conserved catalytic core and a similar overall positioning of the RNA relative to the active site (Fig. 5C), with partially conserved spatial relationships of key RNA-contacting regions (see also Fig. 6 for detailed comparisons). This structural alignment served as the basis for a more detailed comparative analysis of their architectures embedding the 5′SS or 3′SS (Figs 5D and 6), which in turn informed evolutionary insights discussed below. As shown in Fig. 5D, a eukaryotic-specific loop (ESL) is located in human TSEN at a position analogous to the ASL in ARMAN-2 VSEN. This ESL is conserved among eukaryotic TSEN2 orthologs (Supplementary Fig. S7) and occupies a similar spatial position near the 5′SS, suggesting a potentially analogous functional role in RNA recognition and splice-site positioning.

To further elucidate RNA recognition at the 5′SS, we analyzed the local residue interactions in both complexes (Fig. 6A and B). In human TSEN, residue R73 from the ESL of TSEN2 is positioned near the A37 phosphate, and K12 from the KRKRR motif of TSEN2 is located adjacent to the G38 phosphate. Likewise, R279 and W306 of TSEN34 are located in proximity to the bulged nucleotide U36, with R279 positioned in a way that could contribute to the bending of the bulge, a feature comparable to that of R275 in ARMAN-2 VSEN as discussed above. In all available cryo-EM structures of human TSEN, the side-chain density of R73 is weak, and only the main chain is clearly visible in two of these structures [26, 29]. Given this limited local resolution, these descriptions reflect spatial proximity rather than definitive hydrogen-bond or π–cation interaction evidence. Nevertheless, mutational analysis (R73A/K361A double mutant) abolishes intron cleavage activity [29], supporting a significant role for R73 in 5′SS recognition. In contrast, the ARMAN-2 VSEN complex lacks the equivalents of TSEN2 residues K12 and R73, consistent with a less stringent recognition network. Functional studies support these observations: intron-cleavage activity was markedly reduced as the result of alanine substitutions in the KRKRR motif and by the double mutants R73A/K361A, and R279A/W306A [29]. Moreover, the catalytic residues of TSEN2 (Y369, H377, K416) align closely with their archaeal counterparts in ARMAN-2 VSEN, indicating that catalytic geometry is conserved. At the 3′SS, similar conformations are observed (Fig. 6C and D). TSEN54 residue K41 forms hydrogen bonds with the phosphate and 5′-oxygen of A53, occupying a position analogous to TSEN2 K12. TSEN2 residues R409 and R452 participate in π–cation interactions with the bulged nucleotide C51, and R409 also contacts the O2′ of C51 and the phosphate of U54. Although individual R409A and R452A mutations had minimal impact, the combined R409A/K239A/K41A triple mutant abolished cleavage activity, suggesting cooperative function. TSEN34 catalytic residues (Y247, H255, K286) closely mirror the active site geometry of ARMAN-2 VSEN, reinforcing the functional conservation of the catalytic architectures. Unlike the 5′SS, where a long and unstable intron section would require extensive stabilization for catalysis to occur, the 3′SS harbors a canonical BHB-like bulge of only 3 nt. Thus, even minimal stabilizing interactions can suffice for cleavage at the 3′SS, while cleavage at the 5′SS relies on a conformation that depends on auxiliary elements like the ESL. These observations suggest that TSEN, ϵ_2_-type VSEN, and (αβ)_2_-type VSENs from Crenarchaea may have independently acquired structurally analogous auxiliary loops—namely, the ESL, the ASL, and the CSL—through convergent evolution. Despite distinct amino acid sequences, these loops are positioned near the active site and appear to stabilize the bulged region of the intron in cases where canonical BHB motifs are distorted or absent. This convergence likely reflects a shared evolutionary pressure to cleave structurally diverse or destabilized intron substrates with high positional precision. Interestingly, the (αβ)_2_-type VSEN from *Methanopyrus kandleri*, which lacks a defined CSL, exhibits only limited activity toward BHL-type pre-tRNAs [24], emphasizing the functional relevance of these auxiliary features. TSEN may have evolved from a similar ancestral (αβ)_2_-type VSEN, and gradually acquired structural elements such as the ESL and KRKRR motif to enhance substrate specificity and cleavage fidelity at the 5′SS. Moreover, TSEN34 harbors a THUMP domain (Fig. 5A)—a structural module capable of recognizing the mature L-shaped tRNA architecture [26–29]—which likely enables TSEN to act as a structural ruler and stably engage full-length pre-tRNAs [11, 12]. Together with the recruitment of cofactors such as CLP1 [26, 28], these structural and functional changes may have equipped TSEN to meet the complex processing requirements of eukaryotic tRNA maturation. Collectively, these comparative and mutational insights underscore the mechanistic importance of auxiliary loops—such as ASL, CSL, and ESL—in precisely positioning the scissile phosphate and stabilizing the bulged intron region. It is noteworthy that both TSEN and archaeal VSEN appear to exhibit broad substrate specificity, yet they achieve it through distinct strategies: TSEN is thought to employ a two-tiered mechanism involving global pre-tRNA positioning by the THUMP domain and local adaptability via multiple contacts with the bulged intron, including the ESL, whereas VSEN utilizes a more direct interaction between the ASL and the intron bulge, independent of overall pre-tRNA architecture, as shown by our experimental results.

### Mechanistic model for RNA recognition and intron cleavage by ARMAN-2 VSEN

**Figure 7.**
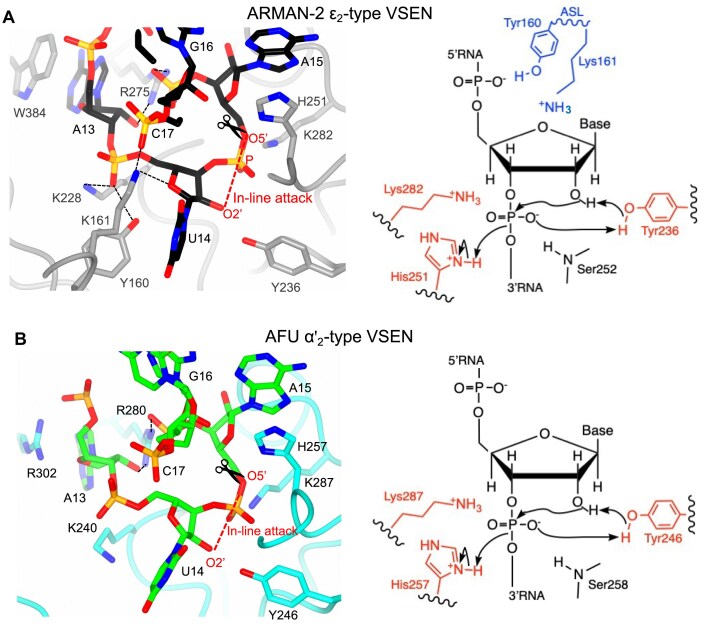
Structural basis and mechanistic comparison of intron cleavage by ARMAN-2 ϵ_2_-type VSEN and AFU α′_2_-type VSEN. (**A**, **B**) Catalytic centers of ARMAN-2 VSEN (PDB ID: 9W2K) and AFU VSEN (PDB ID: 2GJW), respectively, occupied by the 5′SS of the intron. In both structures, the bulged RNA segment is modeled with U14 (replacing dU14) and depicted as sticks colored as in Fig. 2 with carbon in black [panel (A)] or in green [panel (B)]. Key catalytic residues (Tyr, His, and Lys), along with additional residues involved in bulge recognition and scissile phosphate stabilization, are shown as sticks colored as in Fig. 2, with carbon in gray [panel (A)] or in cyan [panel (B)]. Red dashes indicate the proposed in-line nucleophilic attack by the 2′-OH of U14. Right panels: schematic illustrations of the catalytic site environments and electron relay mechanisms during intron cleavage. Both enzymes employ a conserved His–Tyr–Lys triad (in red) but differ in auxiliary interactions: ARMAN-2 VSEN uniquely features ASL residues Tyr160 and Lys161 (in blue), which interact with the ribose O4′ and phosphate of C17 to stabilize the distorted bulge conformation. In contrast, AFU VSEN lacks an ASL and relies solely on conserved residues from the core structure to coordinate the reaction. These mechanistic differences may underlie the broader substrate adaptability of ARMAN-2 VSEN.

Based on the structural and biochemical findings described above, we propose a mechanistic model for RNA recognition and intron cleavage by ARMAN-2 VSEN (Fig. 7). As illustrated in Fig. 7A, the active site at the 5′SS is precisely arranged to facilitate an in-line nucleophilic attack by the 2′-OH of U14 on the adjacent phosphate. Key residues—Y236, H251, and K282—are conserved catalytic components that stabilize the transition state and mediate the cleavage reaction. Surrounding these, additional residues such as K228, R275, and W384 contribute to bulge stabilization and substrate positioning. Importantly, Y160 and K161, located on the ASL, function cooperatively: Y160 appears to stabilize the side chain of K161, thereby facilitating hydrogen bonding between K161 and the ribose O4′ of U14. This interaction is proposed to fix the conformation of the bulged RNA, precisely aligning the scissile phosphate for catalysis. Such geometry may accelerate the rate of in-line attack, enhancing catalytic efficiency. The right panel of Fig. 7A illustrates these key molecular interactions and highlights the electron relay mechanism proposed to underlie intron cleavage. This integrated model not only provides a structural basis for catalysis but also explains how ARMAN-2 VSEN achieves broad substrate specificity. The ASL-mediated RNA engagement offers the conformational adaptability necessary to accommodate diverse intron architectures—such as BHB and BHL motifs—while preserving the fidelity of scissile phosphate positioning. This dual capacity for flexibility and precision likely underlies the enzyme’s ability to cleave a wide range of pre-tRNA introns. Given the shared architectural framework yet distinct auxiliary loops observed in ϵ_2_-type and (αβ)_2_-type archaeal VSENs, we hypothesize that these enzymes may generally cleave introns with higher catalytic efficiency than α_4_- or α′_2_-type VSENs. This could stem from more effective transition-state stabilization and accurate positioning of the scissile phosphate—facilitated by loop-mediated RNA engagement and refined active-site geometry. Further comparative kinetic analyses will be needed to quantify these mechanistic differences. Figure 7B depicts the mechanistic model for the AFU α′_2_-type VSEN, further illustrating the structural and mechanistic differences between α′_2_- and ϵ_2_-type VSENs. Although in both types of enzymes conserved catalytic residues and π–cation interactions stabilize the bulged nucleotides in their substrate, their modes of RNA positioning differ markedly. In ϵ_2_-type enzymes, the ASL loop provides an interaction platform flexible enough that noncanonical bulges can be accommodated and thereby introns with variable conformations. In contrast, α′_2_-type VSENs lack such a structural element which may restrict their affinity to only those RNA substrates that are sufficiently stabilized by interactions involving conserved residues such as R280 and K287. These structural constraints may limit the substrates that can be efficiently cleaved to those that comprise a canonical BHB motif. The absence of an ASL-like loop in α′_2_-type VSENs could thus explain both the reduced substrate flexibility and the evolutionary retention of strict motif specificity observed for this class of enzymes. The comparative models underscore how auxiliary structural features—such as the ASL—serve to extend the substrate repertoire of archaeal endonucleases by enabling precise positioning of the scissile phosphate even in noncanonical or distorted introns. In this context, the ASL not only enhances catalytic geometry but can be understood as an evolutionary change that supports functional versatility.

## Conclusion

Overall, our findings suggest that ASL and its functional analog CSL represent evolutionary changes that underlie the expanded structural and functional repertoire of archaeal splicing endonucleases. The presence of these loops may mirror the diversification of RNA intron structures during archaeal evolution, offering insights into the molecular adaptations that enable broad and efficient RNA processing. While these loops may reflect structural adaptations associated with the diversification of archaeal introns, the possibility that they were retained in certain lineages while lost in others cannot be excluded. These findings may also provide a useful framework for interpreting analogous mechanisms in eukaryotic RNA-processing systems.

## Supplementary Material

gkaf845_Supplemental_File

## Data Availability

The crystal structure factor and coordinates have been deposited in the Protein Data Bank (PDB code: 9W2K).

## Notes

Present address: Computational Medicine Center, Sidney Kimmel Medical College, Thomas Jefferson University, Philadelphia, Pennsylvania 19107, United States
