# Supplementary material for: Structural basis of substrate diversity and functional evolution of archaeal RNA-splicing endonucleases
Source: Nucleic Acids Res. 2025 Aug 30;53(16):gkaf845. doi: 10.1093/nar/gkaf845 (PMC12397907; doi:10.1093/nar/gkaf845)
Supplement: gkaf845_Supplemental_File [file gkaf845_supplemental_file.pdf]

Supplementary data:

Structural basis of substrate diversity and functional evolution of archaeal RNA-splicing endonucleases

Yuna Miyata[1], Ryota Yamagami[2], Takuya Kawamura[2,3], Hiroyuki Hori[2] and Akira Hirata[1,*]

[1] Department of Natural Science, Division of Science and Technology, Graduate School of Sciences and Technology for Innovation, Tokushima University, 2-1 Minamijosanjima-cho, Tokushima, Tokushima 770-8506, Japan

[2] Department of Applied Chemistry, Graduate School of Science and Engineering, Ehime University, 3 Bunkyo-cho, Matsuyama, Ehime 790-8577, Japan

[3] Present address: Computational Medicine Center, Sidney Kimmel Medical College, Thomas Jefferson University, Philadelphia, Pennsylvania 19107, USA

[*]To whom correspondence should be addressed:

Akira Hirata

Phone and Fax: +81-88-656-7261

e-mail: ahirata@tokushima-u.ac.jp

Supplementary Table S1.

List of primers used for the construction of four single mutants (Y160A, Y160F, K161A, K161R), the double mutant (Y160A/K161A) and the deletion mutant (ΔASL).

Y160A

Forward

5´-GACAATACGGGACCGCGAAGGTAAGCGAACAT-3´

Reverse

5´-ATGTTCGCTTACCTTCGCGGTCCCGTATTGTC-3´

Y160F

Forward

5´-GACAATACGGGACCTTTAAGGTAAGCGAACAT-3´

Reverse

5´-ATGTTCGCTTACCTTAAAGGTCCCGTATTGTC-3´

K161A

Forward

5´-CAATACGGGACCTACGCGGTAAGCGAACATGG-3´

Reverse

5´-CCATGTTCGCTTACCGCGTAGGTCCCGTATTG-3´

K161R

Forward

5´-CAATACGGGACCTACCGCGTAAGCGAACATGG-3´

Reverse

5´-CCATGTTCGCTTACGCGGTAGGTCCCGTATTG-3´

Y160A/K161A

Forward

5´-CAATACGGGACCGCGGCGGTAAGCGAACATGG-3´

Reverse

5´-CCATGTTCGCTTACGCGGTAGGTCCCGTATTG-3´

Deletion (ΔASL)

Forward

5´-GGAAACCTAAACAAACTTGATATCTATGAAAC-3´

Reverse

5´-CCCGTATTGTCCAAGCCAAAAAT-3´

The name of the primer is in bold face; mutational sequences are colored red.

Supplementary Table S2.

Protein–RNA interactions in the ARMAN-2 VSEN–RNA complex, including hydrogen bonds, van der Waals contacts, and water-mediated interactions with distances in Å.

Protein Residue	RNA Atom	Interaction Type	Distance (Å)
LYS A 161 NZ	A F 14 O4'	Hydrogen bond	3.04
LYS A 161 NZ	G H 17 OP1	Hydrogen bond	2.91
ARG A 234 NE	A F 14 O2'	Hydrogen bond	3.36
LYS A 282 NZ	A H 15 O5'	Hydrogen bond	3.12
LYS B 282 NZ	A I 15 OP1	Hydrogen bond	3.00
TYR B 160 OH	DU I 14 OP1	Hydrogen bond	2.61
LYS B 161 NZ	DU I 14 O4'	Hydrogen bond	3.00
LYS B 161 NZ	C I 17 OP1	Hydrogen bond	2.92
LYS B 228 NZ	DU I 14 OP1	Hydrogen bond	2.95
LYS A 228 NZ	A F 14 OP1	Hydrogen bond	2.87
TRP B 249 NE1	U I 18 OP1	Hydrogen bond	3.02
SER B 252 N	A I 15 OP2	Hydrogen bond	2.69
ARG A 275 NH1	G I 16 OP1	Hydrogen bond	2.95
ARG A 275 NH2	A I 13 O2'	Hydrogen bond	2.91
ARG B 275 NH1	A F 16 OP1	Hydrogen bond	3.30
ARG B 275 NH2	A F 13 O2'	Hydrogen bond	3.06
TYR A 160 OH	A F 14 C8	Van der Waals	3.14
TYR A 236 OH	A F 14 O2'	Van der Waals	2.93
GLU A 248 O	A F 14 C2	Van der Waals	3.46
HIS A 251 CE1	G H 16 O2'	Van der Waals	3.26
SER A 252 N	A F 14 O1C	Van der Waals	2.49
SER A 252 CB	A F 14 O2C	Van der Waals	3.44
HIS A 278 CE1	C I 11 O3'	Van der Waals	3.33
HIS A 278 CE1	C I 12 OP1	Van der Waals	2.84
TRP A 384 NE1	A I 13 C2	Van der Waals	3.49
TRP A 384 CZ2	A I 13 C4	Van der Waals	3.44
TRP A 384 CH2	A I 13 N7	Van der Waals	3.50
ARG A 234 NH2	DU I 14 C6	Van der Waals	3.37
HIS B 251 CA	A I 15 OP2	Van der Waals	3.01
HIS B 251 C	A I 15 OP2	Van der Waals	3.28

HIS B 251 ND1	A I 15 O4'	Van der Waals	3.13
HIS B 251 CE1	G I 16 O2'	Van der Waals	3.11
HIS B 278 CE1	U F 11 O3'	Van der Waals	3.43
HIS B 278 CE1	C F 12 OP1	Van der Waals	2.82
LYS B 282 CE	A I 15 OP1	Van der Waals	3.18
LYS B 282 NZ	A I 15 P	Van der Waals	3.41
LYS B 282 NZ	A I 15 OP1	Van der Waals	3.00
LYS B 282 NZ	A I 15 C5'	Van der Waals	3.07
LYS B 282 NZ	A I 15 C5'	Van der Waals	3.06
TRP B 384 NE1	A F 13 C2	Van der Waals	3.48
TRP B 384 CZ2	A F 13 O4'	Van der Waals	3.39
TRP B 384 CZ2	A F 13 C4	Van der Waals	3.40
TRP B 384 CH2	A F 13 C8	Van der Waals	3.49
TRP B 384 CH2	A F 13 N7	Van der Waals	3.45

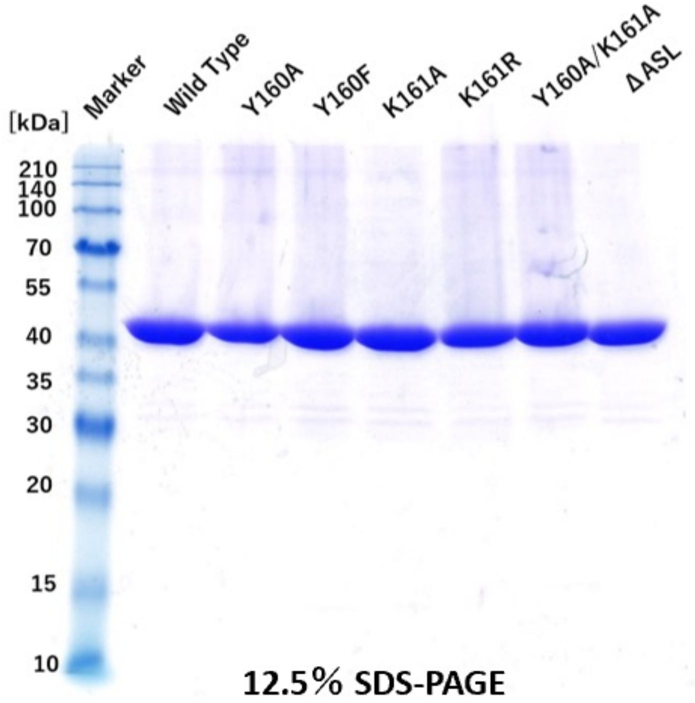

Supplementary Figure S1.

SDS–PAGE analysis of wild-type and mutant ARMAN-2 VSEN proteins. Samples were separated on a 12.5% SDS–PAGE and stained with Coomassie Brilliant Blue. Lane order (left to right): molecular weight marker, wild-type, Y160A, Y160F, K161A, K161R, Y160A/K161A, and ΔASL.

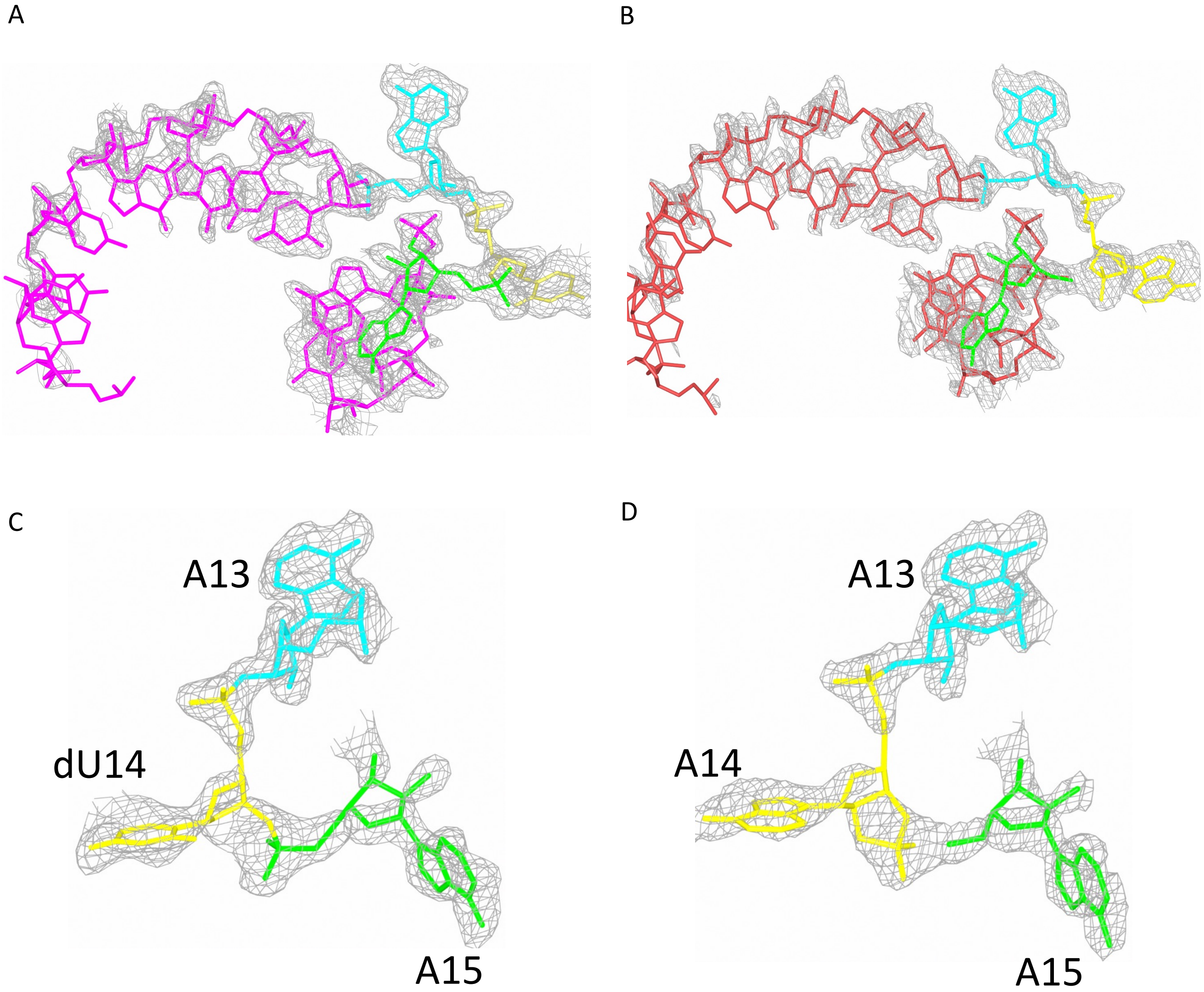

Supplementary Figure S2

Polder omit maps of the RNA substrate calculated with phenix.polder after removal of the RNA coordinates to minimize model bias.

(A, B) Maps contoured at 2.0 σ for single-stranded RNA segments (nucleotides 6–18) encompassing the bulged nucleotides A13–dU14–A15. In (A), the segment corresponds to the 5′ splice site (5′SS) within the BHB motif; A13, dU14, and A15 are shown as cyan, yellow, and green sticks, respectively, with the remaining nucleotides in magenta. In (B), the corresponding segment at the 3′ splice site (3′SS) is shown, with the bulged nucleotides colored as in (A) and the remaining nucleotides in brown.

(C, D) Close-up views of the bulged regions with polder omit maps contoured at 2.5 σ, overlaid with the refined RNA models. (C) 5′SS bulge comprising A13–dU14–A15. (D) 3′SS bulge comprising A13–A14–A15.

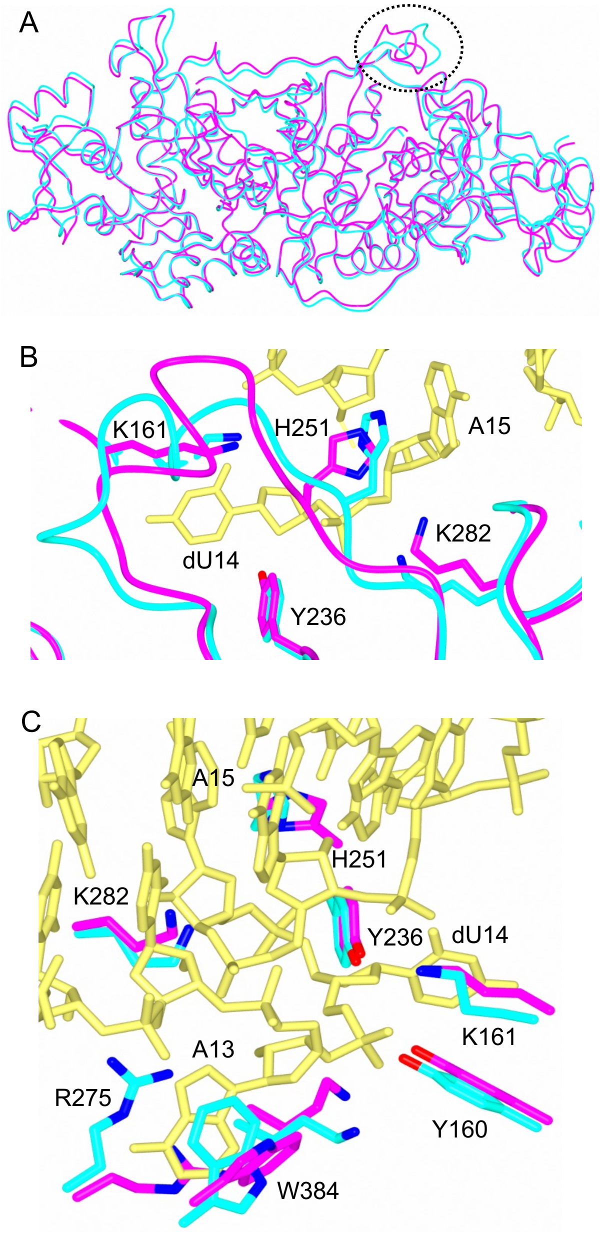

Supplementary Figure S3

Structural comparison of ARMAN-2 VSEN and its RNA-bound complex. (A) Superimposed Cα trace models of the apo-form of ARMAN-2 VSEN and its RNA-bound complex. Structural deviations between the two forms are encircled with dashed lines. The apo-form and complex structures are shown in magenta and cyan, respectively.

(B) Close-up view of the active site around the 5′ splice site, highlighting the bulged region formed by nucleotides dU14 and A15, along with the three catalytic residues Y236, H251, and K282. Residues and RNA are illustrated as stick models, with RNA shown in yellow. (C) Detailed view of the 5′ splice site bulge region including nucleotides A13, dU14, and A15. In addition to the three catalytic residues, K161 and Y160 from the ASL, and R275 and W384 involved in A13 recognition, are depicted. All residues and RNA are shown as stick models, with RNA in yellow.

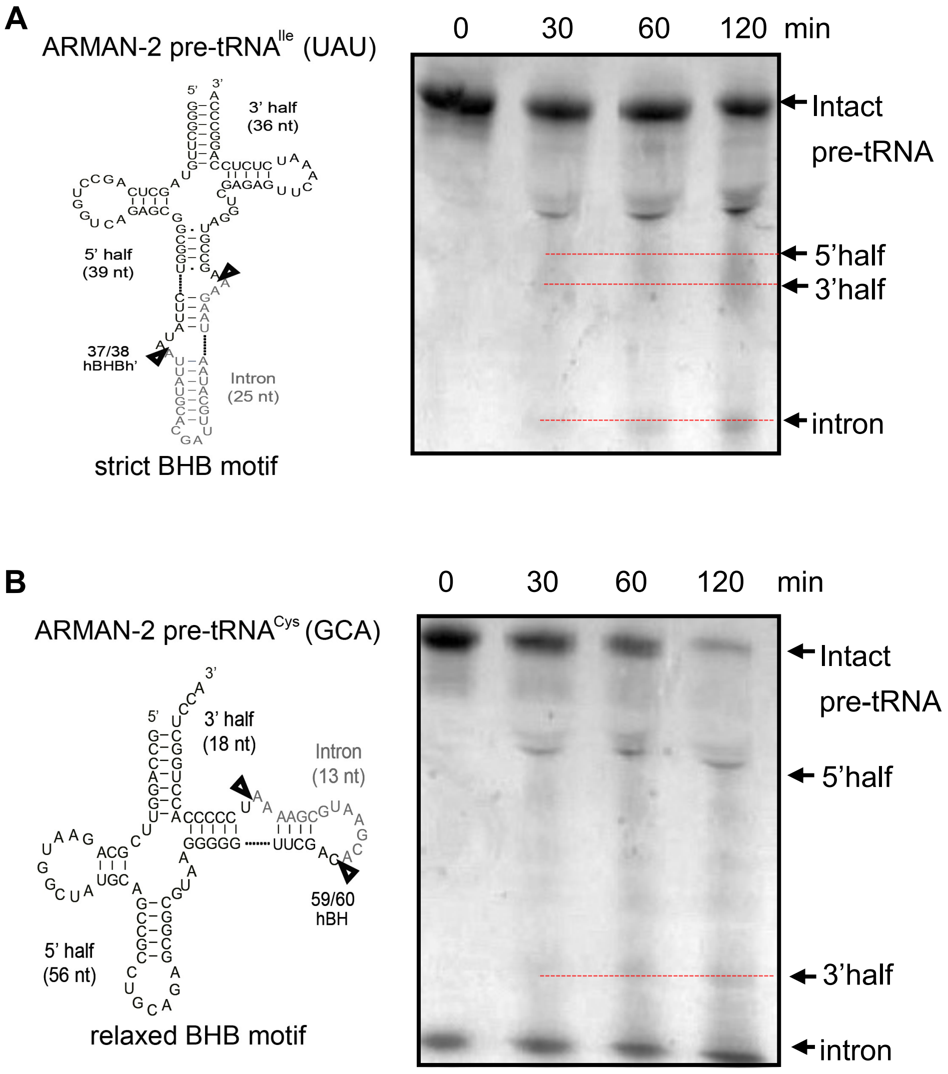

Supplementary Figure S4

Time-course intron-cleavage assay of the ASL deletion mutant (ΔASL) of ARMAN-2 VSEN using two pre-tRNA substrates. (A) Cleavage of pre-tRNA^{Ile} (UAU) containing a BHB motif. (B) Cleavage of pre-tRNA^{Cys} (GCA) containing a BHL motif. Reaction products were analyzed at 0, 30, 60, and 120 minutes by 7 M urea–PAGE and stained with 0.05% toluidine blue. The 5′ exon, intron, and 3′ exon bands are indicated by arrows on the right side of each gel image, with red dotted lines extending from the arrow tips to highlight the corresponding bands.

A

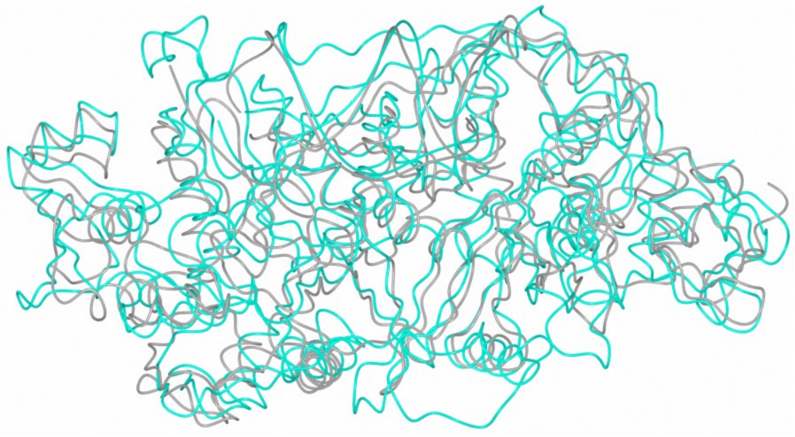

B

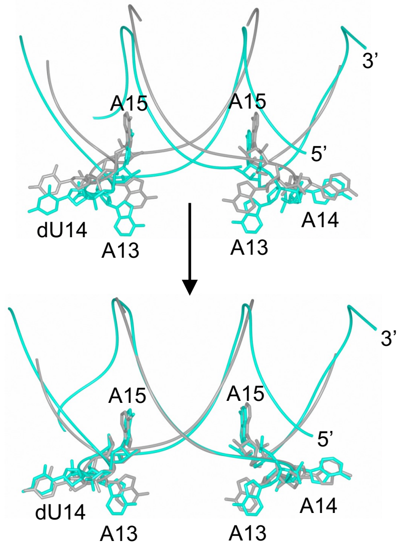

Supplementary Figure S5

Structural comparison of the ARMAN-2 VSEN–RNA complex with the AFU VSEN–RNA complex (PDB ID: 2GJW). (A) Line diagram of Cα atoms of ARMAN-2 VSEN (dark grey) superimposed on that of AFU VSEN (cyan), based on 211 equivalent Cα atoms (RMSD = 3.00 Å). (B) Line diagram of the phosphate backbone of ARMAN-2 VSEN RNA (dark grey) superimposed on that of AFU VSEN (cyan), based on 80 corresponding atoms (RMSD = 0.86 Å). The three nucleotides forming the intron bulge are highlighted in stick representation.

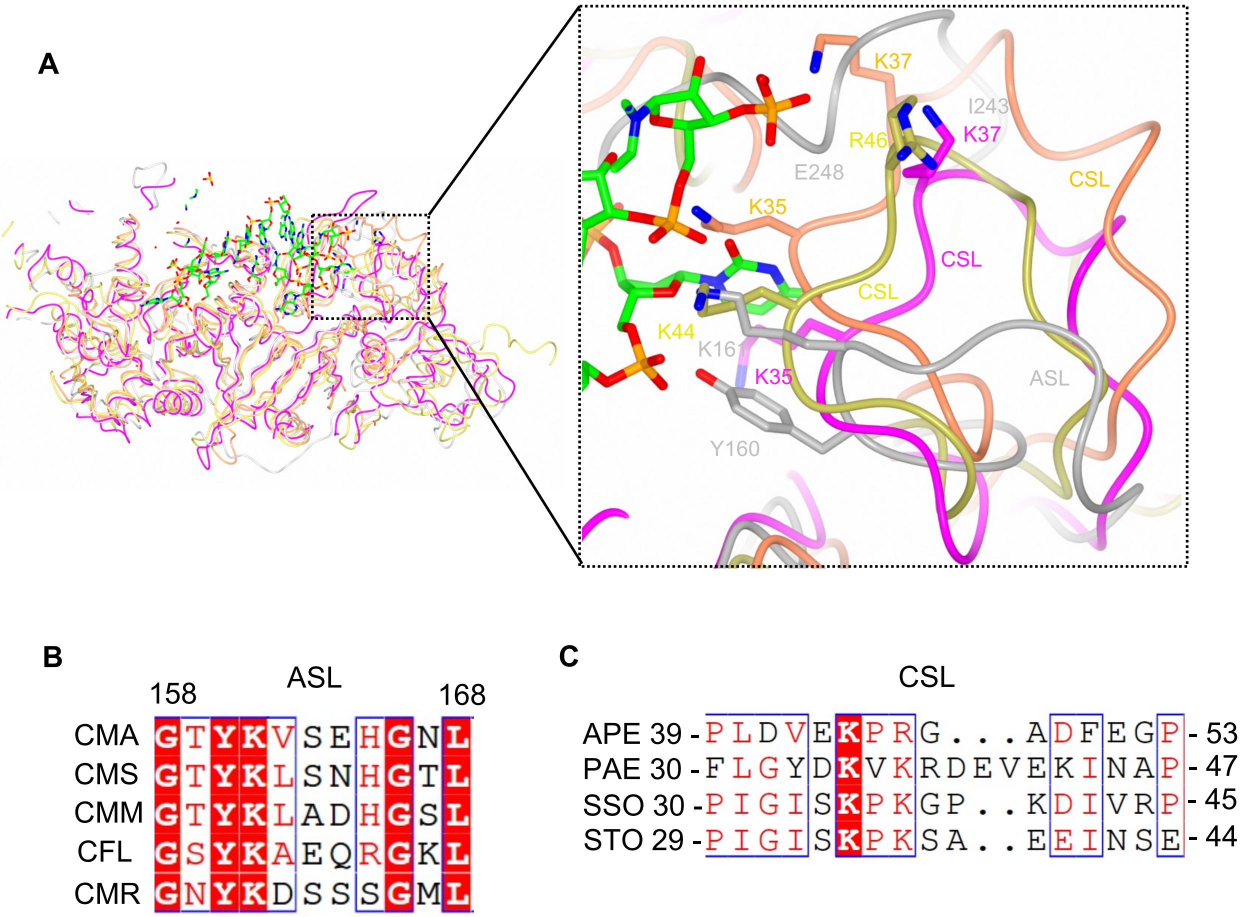

Supplementary Figure S6

Structural and sequence alignment of the ARMAN-specific loop (ASL) and the Crenarchaeal-specific loop (CSL). (A) Superposition of the Cα backbone of the ARMAN-2 VSEN–RNA complex (dark grey, RNA in green sticks) with those of *Aeropyrum pernix* (APE, yellow, PDB ID: 3AJV), *Pyrobaculum aerophilum* (PAE, coral, PDB ID: 2ZYZ), and *Sulfolobus solfataricus* (SSO, magenta, PDB ID: 2CV8). RMSD values for the superpositions are as follows: 2.97 Å over 589 Cα atoms (ARMAN-2 vs. APE), 2.92 Å over 463 Cα atoms (ARMAN-2 vs. PAE), and 2.17 Å over 295 Cα atoms (ARMAN-2 vs. SSO). A close-up view highlights the positions of the ASL and CSL near the catalytic center. The lysine residue K161 in the ASL corresponds spatially to conserved lysines within the CSL. (B) Multiple sequence alignment of ASL regions from ARMAN-2 (CMA), *Candidatus Mancarchaeum acidiphilum* Mia14 (CMS), *Candidatus Micrarchaeota archaeon* ARM-1 (CMM), *Candidatus Fermentimicrarchaeum limneticum* (CFL), and *Candidatus Micrarchaeum* sp. A_DKE (CMR). (C) Multiple sequence alignment of CSL regions from APE, PAE, SSO, and *Sulfolobus tokodaii* (STO). Sequence alignments were performed using ClustalW (50) and visualized with ESPript (51).

TSEN2

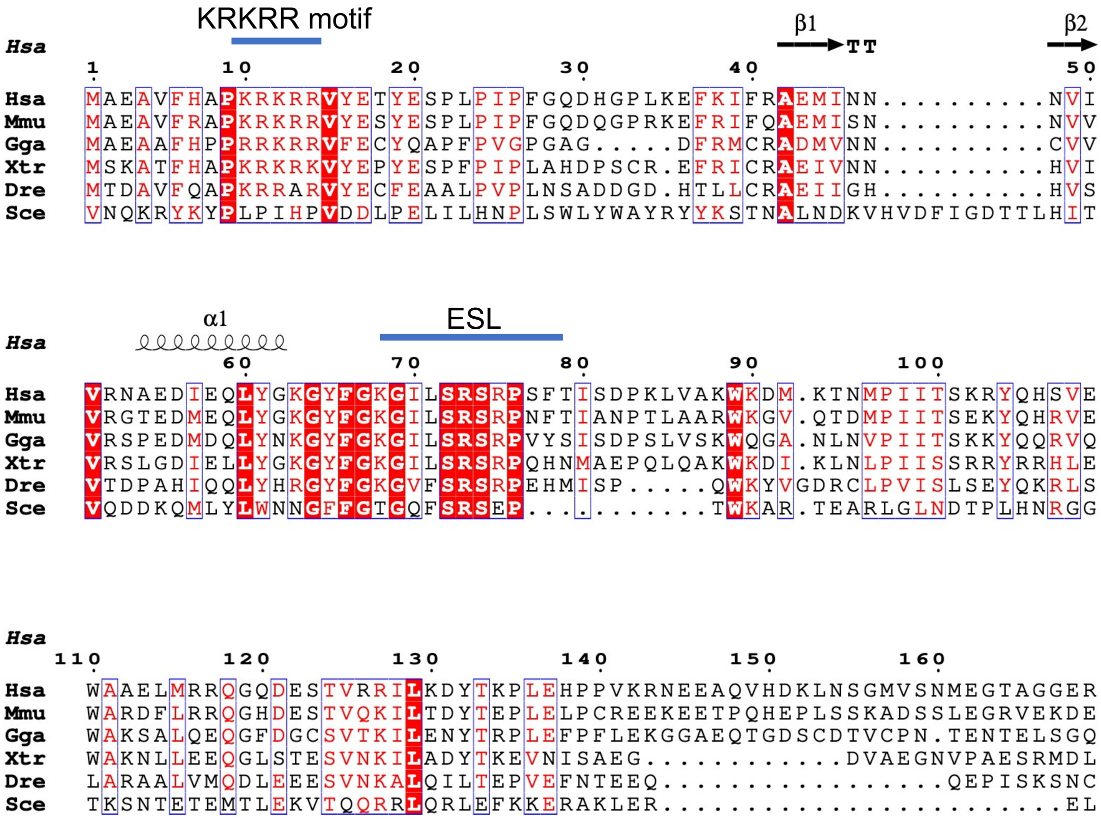

Supplementary Figure S7

Multiple sequence alignment of TSEN2 orthologs from *Homo sapiens* (Hsa), *Mus musculus* (Mmu), *Gallus gallus* (Gga), *Xenopus tropicalis* (Xtr), and *Saccharomyces cerevisiae* (Sce). Secondary structure elements from the human TSEN2 structure (PDB code 8ISS) are indicated above the alignment. The KRKRR motif and the eukaryotic-specific loop (ESL) are highlighted by a blue bar. This figure was performed using ClustalW (50) and visualized with ESPript (51).

References

50. Chenna, R., Sugawara,H., Koike, T., Lopez, R., Gribson, T., Higgins, D., and Thompson, J. (2003) Multiple sequence alignment with the Clustal series of programs. *Nucleic Acids Res*, **31**, 3497–3500. https://doi.org/10.1093/nar/gkg500
51. Gouet,P., Robert,X. and Courcelle,E. (2003) ESPript/ENDscript: Extracting and rendering sequence and 3D information from atomic structures of proteins. *Nucleic Acids Res*, **31**, 3320–3323. https://doi.org/10.1093/nar/gkg556
